# Motivations and barriers to cervical cancer screening among HIV infected women in HIV care: a qualitative study

**DOI:** 10.1186/s12905-015-0243-9

**Published:** 2015-10-12

**Authors:** Agnes Bukirwa, Joan N. Mutyoba, Barbara N.Mukasa, Yvonne Karamagi, Mary Odiit, Esther Kawuma, Rhoda K. Wanyenze

**Affiliations:** Mildmay Uganda, Kampala, Uganda; Makerere University School of Public Health, Kampala, Uganda

**Keywords:** Cervical screening, Uptake, HIV positive women, Integrated HIV/ SRH services, Barriers, Motivators

## Abstract

**Background:**

Cervical cancer is the second commonest cancer in women worldwide and the commonest cancer among women in Uganda. Annual cervical screening is recommended for women living with HIV for early detection of abnormal cervical changes, however uptake remains grossly limited. This study assessed factors associated with cervical screening uptake among HIV infected women at Mildmay Uganda where cervical screening using Visual inspection with acetic acid and iodine (VIA and VILI) was integrated into HIV care since July 2009.

**Methods:**

Eighteen (18) in-depth interviews with HIV infected women and 6 key informant interviews with health care providers were conducted in April 2013 to assess client, health care provider and facility-related factors that affect cervical screening uptake. In-depth interview respondents included six HIV infected women in each of the following categories; women who had never screened, those who had screened once and missed follow-up annual screening, and those who had fully adhered to the annual screening schedule. Data was analyzed using content analysis method.

**Results:**

Motivations for cervical cancer screening included the need for comprehensive assessment, diagnosis, and management of all ailments to ensure good health, fear of consequences of cervical cancer, suspicion of being at risk and the desire to maintain a good relationship with health care workers. The following factors negatively impacted on uptake of cervical screening: Myths and misconceptions such as the belief that a woman’s ovaries and uterus could be removed during screening, fear of pain associated with cervical screening, fear of undressing and the need for women to preserve their privacy, low perceived cervical cancer risk, shortage of health workers to routinely provide cervical cancer education and screening, and competing priorities for both provider and patient time. Major barriers to repeat screening included limited knowledge and appreciation of the need for repeat screening, and lack of reminders.

**Conclusions:**

These findings highlight the need for client-centered counseling and support to overcome fears and misconceptions, and to innovatively address the human resource barriers to uptake of cervical cancer screening among HIV infected women.

## Background

Cervical cancer is the commonest cancer among women both immune competent and immune compromised, and the leading cause of all cancer related deaths among women worldwide. The burden of cervical cancer is highest in sub Saharan Africa (almost twice the global burden) and even higher among HIV infected women [1–6]. Cervical cancer is also more aggressive in the HIV infected women, thus the recommendation to integrate cervical screening in routine HIV services [6, 7]. Uganda has the highest burden of cervical cancer in the East African region, with an incidence of 22.6 % compared to the regional average of 20.1 % and 15.8 % worldwide [8]. Cervical cancer related deaths are also highest in Uganda at 15.6 % compared to 13.8 % for East Africa and 8.2 % worldwide [8].

Despite the high global burden of cervical cancer, cervical screening uptake remains poor, especially in the developing countries [1, 9]. Inadequate knowledge about the disease and its prevention as well as long distance to the screening sites are some of the factors influencing uptake of cervical screening services [1, 10]. Other factors include the need for partner’s approval, negative attitude of clients towards the service, and socioeconomic factors as well as health care system constraints [1, 10–12].

“For the HIV infected women in care, studies from Africa and elsewhere have demonstrated that integrating HIV and sexual and reproductive health (SRH) services may benefit women by improving their unmet need for a range of reproductive health services including contraceptive use and condom use [13, 14], and enhanced user satisfaction [15, 16]. Integrated SRH and HIV services may similarly strengthen cervical cancer screening, which is increasingly offered as part of the SRH care package. Parallel services present challenges of transportation access, inconveniences of setting up other appointments for screening etcetera [17]. Integration of cervical screening services within HIV care could thus potentially overcome such barriers [18] and increase uptake of cervical screening services.

In 2009 Mildmay Uganda integrated cervical cancer screening into HIV care and treatment services. However, as documented elsewhere, there has been persistent low uptake of cervical screening despite the integration of services [19]. Several gaps and questions remain in terms of in-depth understanding of why high-risk women should fail to screen for cervical cancer even after integration of this service into their routine HIV care. Do the women appreciate the value of screening; are they willing to undergo screening; is the screening process done in a manner that enhances the likelihood of screening? The purpose of this study was to document factors affecting uptake of cervical screening among HIV infected women receiving care at a facility that provided integrated HIV and SRH services including cervical cancer screening.

## Methods

This cross-sectional qualitative study was conducted in April 2013. Eighteen in-depth interviews (IDIs) were conducted with adult women (25 years and older) in HIV care at the Mildmay HIV clinic. The IDI respondents included women who had never screened for cervical cancer (6 women), those who had screened only once and missed subsequent screening (6 women), and those who had fully adhered to their annual cervical screening schedule (6 women). Six purposively selected health workers, across various clinic departments, were also interviewed as key informants. The key informants included two nurses from the cervical cancer screening unit, one nurse from the nurse-led HIV clinic, one clinician from the adult HIV clinic and one from the paediatric/family clinic (a clinic that attends to children with their parents), and one “expert” client (HIV infected client who has been in care for a long time and has exemplary practices in relation to HIV service health seeking behaviors).

### Study site

Mildmay Uganda is an HIV specialist care organization situated 12 KM from the capital city of Kampala, in Uganda. Mildmay offers HIV services with integrated sexual and reproductive health (SRH) services including cervical screening using Visual Inspection with Acetic acid or Iodine. The program integrated health education related to cervical cancer screening in the daily morning health talks that are delivered in the mornings to clients in the waiting area. Since 2012, cervical cancer related health education was also given in the clinicians’ rooms and clients offered a chance to screen. Early lesions are treated with cryotherapy while advanced cervical cancer cases are referred to higher-level specialized facilities.

### Data collection, management and analysis

Key informant interviews (KIs) and IDIs were carried out with purposively selected respondents using interview guides that were customized to the different categories of respondents. For the women who had never screened, the themes included knowledge about cervical cancer, need for screening, how often they should screen, and risk perception, among other issues. They were also asked directly why they had never screened and whether a provider had ever talked to them about screening. Those that had screened only once were also asked questions around knowledge and risk perception, schedule for screening, and they were asked directly about why they had not gone for repeat screening as well as hindrances to repeat screening. For the women who adhered to the screening schedule, the questions addressed their experience with the screening and motivators for screening (Table 1). The in-depth interview guides were translated into Luganda, the commonest local language for the study setting while the KI guides were administered in English. IDI and KI interviews were audio-recorded and transcribed verbatim. For the IDIs translation from Luganda to English was done concurrently with transcription. The interview guides were pre-tested at Midmay Uganda; the research assistants interviewed one client in each of the three categories (never screened, screened once, and adherent to screening schedule) as well as one key informant to ensure appropriateness of the questionnaires to the targeted groups. The data for these pilot interviews was not included in this analysis. Daily meetings were held during data collection to address emerging issues. The data was manually analyzed using the content analysis method. Predetermined themes based on anticipated issues such as barriers, motivators, knowledge, and risk perception were developed in addition to emerging unanticipated themes. Relevant and special verbatim quotes were selected to support the various themes and sub-themes.

**Table 1:** Summary of questions asked to different categories of study participants

Theme	Never screened	Screened once	Screened on schedule
Knowledge about cervical cancer and risk perception	According to what you know, which kinds of people are likely to get the disease? Why do you think that such people are likely to get cervical cancer?	Identical questions	Identical questions
	What are the chances that you can get cervical cancer? Why?		
	How would you grade your chances of getting the disease relative to that of women who are HIV negative and why?		
Disease management	How is cervical cancer treated?	Identical questions	Identical questions
	According to what you know, how can cervical cancer be prevented?		
	How often should women check for cervical cancer?		
Screening uptake	Have you ever been screened for cervical cancer? If no why haven’t you been screened?	I understand that you have ever checked for this cancer at least once, what prompted you to go for the check -up then?	I understand you have been coming for cervical cancer checkup every year, what motivates you to continue coming for the screening?
		*Probe: was it after referral* How come you checked once and have never done it again?	Are there times or factors that sometimes make you feel like you should not go for screening again? If yes mention them. How do you overcome these barriers?
		*Probe: was it the experience, the time etc.? Do you think it is possible to overcome these barriers? If yes how? If no, why?*	
		*Do you still desire to have another checkup? Why do you think women should have repeat screening?*	
Experiences with screening	n/a	During the time you went for the check at Mildmay, what was the experience like?	How is the check-up /screening experience in terms of the procedure/what is done during the check-up?
		*Probe: How was your experience in terms of the procedure/what was done to you during the check?*	*Probe: what did you not like about the procedure?*
		*Probe: what did you not like about the procedure?*	Would you consider the check-up/screening beneficial? Explain
		Would you consider the check/screening beneficial? If yes, in what way?	
Assessing Unmet need and how it can be addressed	Have you ever desired to go for the check-up/screening but failed to access it? What was the hindrance?	What needs to be done or improved to serve you better in the prevention of cervical cancer?	What needs to be done or improved to serve you better in the prevention of cervical cancer?
	Has anyone ever shared with you about this service? Who was this person/s? *What did this person/s tell you about the service? Did they refer you for the* service?		
	*Probe: Did you actually go for the service after referral? If not, why? If yes, did you get the service? If you did not get the service, what made you fail to get the service?*		
	What should be done to facilitate or motivate you to go for screening/check-up?		

## Ethics statement

The ethics and research committees of Makerere University School of Public Health, Mildmay Uganda and the National Council of Science and Technology approved the study. Written informed consent was obtained from all study participants and interviews were conducted in a private setting.

## Results

The factors affecting uptake of cervical screening among HIV infected women at Mildmay Uganda are categorized in six major themes; risk perception, barriers to access, availability and access to information on cervical cancer and related services, facility factors such as space and human resource, motivation for screening and screening experiences for individuals as well as experiences shared by peers.

### Client related factors

#### Risk perceptions for cervical cancer

All the women including those who had never screened knew that they were at high risk of acquiring cervical cancer. They mentioned two factors that increased their risk of acquiring cervical cancer: 1) They were HIV infected implying that they had a lowered immunity and could easily contract any infection; and 2) They were still sexually active and could get infected through sexual exposure.

*“Yes because I am HIV positive and I have a husband who I don’t know where and how he moves so anything can happen” (Client, screened on schedule).*

*“I can get it because may be my immunity is low, but even the one who is negative may get it, may be my chances of getting it are high compared to that one who is not HIV positive” (Client, never screened).*

The knowledge of increased risk of cervical cancer among HIV infected and sexually active women “generic risk perception” was at variance with the individualized risk perception that would drive cervical screening. Whereas all categories of participants knew the value of screening early, some participants still waited for a warning sign like an abnormal vaginal discharge to trigger the screening. Even those that screened did not perceive themselves at risk until they had a warning sign.

“*…I have not really had signs to show me that I might have it” (Client, never screened).*

*“…I had pain in the back for such a long time and…. for many years, I got something that would come out in the vagina; that is why I decided to come here for screening” (Client, screened on schedule).*

*“I went for a checkup it is because I had started developing sign….” (Client, screened on schedule).*

Despite of the similarity in knowledge about cervical cancer across the three groups and close to similar risk perception, there were differences among the never screened and the screened category (both screened once and screened on schedule) in terms of the views held under the other thematic areas that are presented below.

#### Myths and misconceptions about the process of screening

Not knowing how the screening procedure is done seems to generate a lot of speculation about what happens during screening. Among the myths and misconceptions that prevented women from screening was the perception that, health workers were removing ovaries, cutting off pieces of flesh from the person and sometimes completely removing the uterus.

*“…some young girls say that there is something they want from them… but then I heard someone saying that they remove the ovaries” (Client, screened on schedule).*

*“….Some of them are told that the womb is removed and put aside during the checking process. Others say they cut off some piece of flesh……” (Client, screened once).*

The health workers were aware of some of the myths. They also mentioned rumors amongst the patients.

*“…people associate cancer screening with the cutting off of a piece of flesh from the private parts……” (Health worker).*

The women that had never screened also feared pain during cervical cancer screening and other side effects of the procedure. These fears were perpetuated by other women in the clinic.

*“There are certain things they [other patients] tell you and you get scared. They told me that when you go for the checkup you can continue having an abnormal discharge for a period of one month. I know of a lady who they checked and she said it had taken her one month with that abnormal discharge” (Client, never screened).*

*“…the problem that a certain woman got, it scared me too much, after she underwent that screening, her womb was tampered with until she was operated upon” (Client, never screened).*

Those women that had ever screened noted that before the screening they had these same fears but did not experience these problems when they eventually screened.

*“I have not found any problems with the checkup procedure… … for the first time I feared the machine being inserted inside me, but I didn’t find any problem with the machine. (Client screened on schedule).*

#### Poor health status of the women

Those who had never screened cited ill health as a hindrance to cervical screening. Some of the conditions cited were skin rash and excessive weight loss that made the clients uncomfortable to expose their bodies to the health workers. Other illnesses such as diabetes and hypertension were reported to have deterred them from cervical screening due to fear of the stress that would arise from an additional diagnosis of a serious illness.

*“… I feared to add on another issue that would scare me. My skin was looking bad and I had even lost weight. Even my blood pressure was high. So I feared that in case I went for screening and they told me I have it [cervical cancer], how will I be like. It would have frightened me and maybe even get a stroke from there.” (Client never screened).*

While those that had never screened feared another bad diagnosis, those that had screened were interested in getting every disease diagnosed and handled early.

*“I already have HIV and I am a widow, I have to come and check because I don’t want to add another disease so that I can be able care for my children” (Client, screened on schedule).*

#### Competing health priorities and low prioritization of cervical screening

The women who had never screened reported that they had other health problems that they thought were more important to attend to first before cervical cancer screening. Among these were: tuberculosis treatment and taking their antiretroviral drugs.

*“…in case it [cervical cancer] required treatment, I did not want to combine many treatments at the same time even if I was to be found with it. …. by the time I got to know about cervical cancer, I was on TB prophylaxis for six months” (Client, never screened).*

*“I am not yet ready, because I have other critical things that I’m treating now, but I know, I will check… I was strong, but my skin was very bad, so I didn’t think about cancer first. I wanted to take my drugs to have my immunity improved first, but I knew that I would check for cancer also, but first I cared so much about taking the ARVs then I would begin on others like checking for cancer” (Client, never screened).*

On the contrary, those that had screened were comfortable handling all the problems concurrently. They screened because they desired total good health and felt that knowing that they had the disease early would save their lives. They felt the health workers’ advice was always the best and also wanted to be in harmony with health workers.

*“What motivates me is that I want to know if I have that cancer of the mouth of the womb so as to save my life and to continue with my life in future. I also want to move together with my health workers…Now if the health workers tell you that they are going to check you up and you tell them to wait yet they are the ones treating, you will be giving them a hard time” (Client, screened on schedule).*

The women who had never screened also cited being in menstrual periods and being pregnant as hindrances to cervical screening.

*“I used to come when I have just gone out of my menstrual periods and yet they say they don’t want clients who have just gone out of periods. It is the health workers who were telling us that if one has just been in her menstruation period, you should not come.” (Client, never screened).*

However, when probed further, they noted that sometimes it was the long waiting time or complacency that made them postpone the screening.

*“Sometimes the queue is long and yet you came from duty. The other issue it seems “bugayavu” (laziness). So you keep on postponing it” (Client, never screened).*

#### Fear of invasion into their privacy

The fear of invasion into ones privacy was cited as one of the key factors that prevent women from screening for cervical cancer. Many women seemed uncomfortable with “exposing” themselves to health workers during screening. This fear was attributed to a number of reasons including among others; shyness, poor hygiene and disease conditions that women may not want to expose.

*“Again issues to do with private parts are sensitive, most people feel shy talking about them. You know she will say the health worker is going to see my private parts, how will they see that I am infected with this; that is why you find someone has been coming to the clinic for two years but the health worker has never known about her problem” (Client, never screened).*

*“Basically when I was talking to some of them, some of them are very shy to show their private parts. Some of them are attached to the clinicians, they know that every other day they will be coming in and since you have screened…, they think that you can remember that so and so looked like this so that is one of the fears in our clients” (Health worker).*

The fear of undressing was a prominent issue even among the women who had previously screened and this was probably worsened by the screening rooms that were crowded with other SRH services and at the same time very close to the rooms used for other clinical services which may further compromise the privacy. They noted that only those who appreciated the importance of screening broke through this barrier. These concerns were even more prominent for the older women and especially when dealing with the younger health workers.

*“I didn’t find many problems the only problem I got was when they told me to remove my clothes but later I realized they were fellow women. I looked at them, I became strong and I removed the clothes…” (Client, screened once).*

*“Also another issue is about undressing yourself to young health workers…because today I have been with some women at the clinic and they were saying “like me I finished producing long time ago and now how can I expose my privacy to a young person for screening, I got tired of such, that is why I even stopped producing…” Then the other thing is that others come when they are not prepared when their hygiene is poor” (Client, never screened).*

### Facility/process factors

#### Inadequate health education about cervical cancer prevention and management

All categories of women including those that had never screened had basic information about cervical cancer; they were aware of the disease and that it can be prevented and treated. Sixteen out of the eighteen women who were interviewed had heard about cervical screening and cervical cancer from the health education offered at the Mildmay facility.

*“Yes I have ever heard that cervical cancer is one of the sexually transmitted diseases, so it is reasonable to screen for cervical cancer… if you test early enough…… it becomes easier to treat it. If they find that you don’t have it, they advise on what preventive measures you have to take.” (Client never screened).*

Despite the fact that majority heard about cervical cancer from the health education at Mildmay, there were complains about inadequate health education. Almost all women across all categories reported that health education was not readily available, had gaps and was not well-structured. For example majority of the women reported that the time given to health education talks was very short and as such many issues about cervical cancer were not adequately explained to clients. They noted that health workers only inform clients about existence of cervical screening services and encourage them to screen without giving regular and detailed information about the disease, related services and importance of these services. The clients felt that this was due to the heavy work load coupled with inadequate staffing.

*“More so these health talks that are conducted here at the clinic are in intervals, so if someone came very early, she can get some health talks and miss out the others…, you find yourself not knowing.” (Client never screened).*

*“… the health workers just told us that women should go and get checked but they did not explain much about this cancer, they didn’t go into those details, like I told you so I found myself pregnant and I did not know whether it was fine to screen while still in that condition” (Client, never screened).*

Some women who had previously screened did not know that they had to screen again and how often. Those who adhered to the schedule felt they had not been given a good reason why they needed to screen again; they just did it routinely as part of the care package.

*“For me I get checked but there is nothing they tell me that maybe I have the disease, sometimes I have that feeling that maybe I shouldn’t go back another time” (Client screened on schedule).*

*“Irregular screening could be tagged to the inadequate information that we give probably like you screen someone and you tell them come back next year for screening about this time, but you don’t tell them the rationale as to why they need to screen again because you want to beat the line” (Health worker).*

All the women, including those who had ever screened and interacted with the health workers, lacked information on how the disease is treated.

*“I don’t know how cancer of the cervix is treated, maybe they give tablets or maybe they give injection, or they just place them there, I really don’t know… they told me when I went for checking that if it is found that you have it, you will be given some drugs, but for me I don’t know anything about that drug, I don’t know how it is treated” (Client, screened once).*

The six service providers also concurred with the clients that health education about cervical cancer was not adequate. They particularly reported that the timing of the health education did not give opportunity for all clients to receive the information. There are many topics and issues to deal with during education, at different intervals. The health education is also carried out only in the morning and the women who come later miss it. They also reported that health education about cervical cancer was not regular and was not given enough time to address all issues, due to the heavy work load.

*“I would say it is not done on a regular basis depending on the number of health workers because not all health workers are well versed with information on this cancer so we cannot just pick anybody to come and speak about this… so when we are few health workers in the reproductive health then the health talks will not take place (Health worker).*

#### Lack of a proper follow-up mechanism

There was general lack of reminders and information on the screening schedule in the health education. Unlike those who were screening on schedule even without reminders, lack of reminders was a big factor that hindered cervical screening among those that screened only once. Service providers too raised the issue of clients forgetting the due dates for the next screening.

*“No, there is no problem*, *the mistake I made, I did not ask the health worker about how long I should take to be able to come back, and I left the room without asking her… I will check at one point. I know, the benefit of repeating, even if I did not have it the other time, they can find that I have it now” (Client, screened once).*

However, some providers noted that these issues had been identified and there were efforts in place to ensure that clients are reminded to have their repeat screening.

*“…each client has a file and those files are clearly labeled so the health workers are also very vigilant, they will always look at the dates because they are tagged” (Health worker).*

*“At the moment, there is a new system where they have cards, they write for you the next appointment, so the patient keeps it in mind…” (Health worker).*

#### Long waiting time

Long waiting time was another key barrier to cervical screening mainly mentioned by those women that had never screened at all. Some of the women had not screened because they come to the clinic when they have other obligations at home and their work places yet the waiting time for screening at the clinic appears too long. These factors were also reported by service providers in the KI interviews.

*“….. Sometimes you come when you need to go back quickly for work and yet at screening you have to wait for a long time. Sometimes the queue is long and yet you came from duty so you keep on postponing it, but it is still the issue of time” (Client, never screened).*

*“Then others talk about time because that day they have to receive other services. You see one going in for counseling, she also has to go in for blood tests, so at times, they find that the waiting time is too much, and then they postpone to some other day…” (Health worker).*

#### Inadequate space

In all the six KI interviews, service providers noted that limited space for cervical cancer screening was a major problem. The screening rooms were few and crowded with other SRH services. This leads to long waiting time and missed opportunities for screening.

#### Personnel problem

The personnel challenges included low staff numbers as well as poor staff attitude towards cervical screening. All the service providers acknowledged that there was inadequate staffing for cervical screening. This was mainly caused by two issues: few staff were trained in cervical cancer screening and thus the task overload as the same staff who do cervical screening are also involved in providing other health services at the clinic. Because of the few staff available for screening, the health workers found it hard to do screening for all willing clients, thus creating missed opportunities for cervical screening among clients. Apart from staff being few, one service provider reported that given the nature of the work, some staff with skills in cervical screening were not interested in doing the work.

*“There are few health workers who were trained in cervical screening, but then there are some who trained but are not interested because of the nature of the work…. (Health worker).*

#### Logistics

Service providers reported that though not a big problem, sometimes they are faced with stock out of certain supplies such as screening reagents, and inadequate equipment like screening beds and lights.

*“….and I think the equipment, so they are probably rescheduled because like there are few beds and the women have to wait and I think some of them at times are like I can’t’ wait this longer” (Health worker).*

## Discussion

This study assessed barriers and motivations for cervical cancer screening among HIV infected women attending a clinic that had integrated cervical screening. The findings show that the women who had never screened and those who had screened had knowledge about the risk of cervical cancer among HIV infected women and appreciated the need to screen. However, despite this knowledge the triggers for testing among women who had done so were symptoms which they thought were related to cervical cancer. For those that had never screened, major hindrances included various fears and misconceptions about the screening procedures. On the other hand, failure to appreciate the need for repeat screening and lack of reminders were major barriers for those that had screened only once. Other barriers to screening included health system barriers such as inadequate information and staffing. Table 2 summarizes the emerging themes across the three categories of women interviewed.

**Table 2:** Summary of the emerging themes and sub-themes across various categories of women interviewed

Themes	Never screened	Screened once	Screened on schedule
Knowledge and information about the disease and related services	Knew the disease can be prevented and treated.	Same issues as in the never screened group	Same issues as in the never screened group
	Knew that early diagnosis and early treatment is important in management	Same as in the never screened group	Same as in the never screed group
	All were aware of cervical screening as one of the methods of prevention but did not know how often they needed to screen	Same issues as in the never screened group	Same as in the never screened group
	All lacked information on the available methods of treatment	Same as in the never screened group	Same as in the never screened group
.Risk Perception	All knew they were at high risk because of their HIV status and felt susceptible because they were sexually active	Same as in the never screened group	Same as in the never screened group
	However, they did not feel they were immediately at risk because they had not experienced suspicious symptoms	However, some felt they were not at much risk as such because they had not experienced symptoms	Some screened as part of the routine tests while others felt at greater risk because they had experienced suspicious symptoms “a warning sign”
Barriers to screening	Fear of side effects	Other conditions: menstrual periods, pregnancy	No issues raised against repeat screening but some had concerns about feedback after screening and long waiting time
	Poor health e.g. severe wasting and bad skin conditions which they could not expose to health workers	Forgetting due date for next screening and lack of reminders	
	Having more important health priorities (low prioritization of cervical screening over other services)	Not clear about schedule and reasons for repeat	
	Other social/family priorities; lack of time for screening Other conditions: menstrual periods, pregnancy, poor hygiene	Concerns about adequate space and privacy	
	Myths and misconceptions from other clients: providers remove ovaries, flesh, and uterus during the screening		
	Fears: invasion into one’s privacy; fear of undressing; an additional bad diagnosis on top of existing diseases		
	Facility issues: long waiting time, inadequate education		
Motivation for screening	The only reason for screening was the generic perceived risk due to HIV status and being sexually active	Suspicious symptoms	Findings identical to those who screened once
		Being HIV positive, sexually active, and at higher risk	
		Seek treatment/ensure protection	
		Maintain a good relationship with the health workers	
Experiences with screening	Cited experiences shared by those that underwent screening (negative issues presented above)	The experience was satisfying, painless	The experience was satisfying, painless
Cervical cancer screening education	Health education is not informative enough and poorly structured limiting accessibility	Same as in the never screened group	Same as in the never screened group

One of the barriers to uptake of cervical screening in the general population has been distance to the screening sites [1, 10]. This study shows that even where the issue of distance to the screening site has been bridged by providing integrated cervical screening and HIV services several barriers to screening still persist either due to organization of services or client fears and myths around the screening procedures. Another study conducted in Kenya showed that despite frequent contact with the health care system, HIV infected women had poor screening behavior compared to their HIV negative counterparts (19 % of HIV negative women screened compared to 11 % of the HIV infected women) [19]. In another study in Ukraine, only 30 % of HIV infected women had received at least one screening test as part of their HIV care [20].

From the interviews carried out in this study, generally, there was willingness to screen among all client categories, which is in agreement with what Oliver and colleagues found among HIV infected women in routine care in Nigeria [21, 22]. Yes, the will is there and the service is closer to this population but uptake was still low. Our study raises a number of fairly basic issues and concerns that providers need to be aware of and address during health education sessions. Women need to understand how the procedure is done, why they should do it every year when they had negative results, and what will happen to them if they are diagnosed with cervical cancer. The providers also need to be conscious of certain concerns and fears even for potentially mundane issues such as not wanting to undress in front of a health worker and preference for certain categories of health care providers e.g. older women who had challenges with undressing in front of younger providers.

One of the key issues raised by the women was inadequate understanding of the disease and related services including the screening schedule. In this study, many participants who had not screened on schedule were willing to screen but were not sure how often and when to come back. This was partly attributed to limited health education. Although not carried out in an integrated setting, a study in Norway revealed that most of the women who were screened opportunistically were less likely to be aware of the screening schedule and had little knowledge about the disease and related services, compared to those screened regularly [23]. Within the integrated HIV-cervical screening setting, the lack of clear appointments for repeat cervical screening in relation to other appointments for HIV related services may signal to the women that the repeat cervical screening is not as important as other HIV related services. Routine reminders and appointments for screening instead of relying on the patients to initiate the process would address this barrier.

Whereas early diagnosis and treatment is critical in the management of cervical cancer, women who had screened in this study were prompted by suspicious symptoms. Symptoms as a trigger for cervical screening, has been reported in other studies and needs to be addressed in order to attain earlier and sustained cervical screening by HIV infected women [21, 23]. Having many competing health priorities, fear of a positive result or another diagnosis on top of HIV also contributed to low uptake of cervical screening. As reported elsewhere, participants preferred to deal with one or a few issues at a time and to work on what they felt was priority like taking ARVs and anti-Tuberculosis drugs [24]. The motivations for screening such as the need to diagnose and treat all illnesses in order to maintain good health as reported by some women could be emphasized by providers while allaying fears of an additional diagnosis and the myths and misconceptions held by the women.

The women had a number of myths and negative stories about cervical screening; they feared that health care providers would remove their ovaries, cutoff some flesh from them and remove their uterus during the procedures. The fear of pain during the procedure was also prominent. However, the participants who had screened before felt that there was little discomfort if any and the exposure was for a very short time and they were encouraged by the fact that the screening team was made-up of fellow women. Similar findings were reported from a study by Rositch and colleagues in Kenya [19] where over 70 % of the clients felt that the pain associated with screening was minor. Given that this was a prominent barrier, integrating testimonies of the women that have been through this process during health education sessions could counter the negative myths perpetuated by clients and also alleviate the staffing concerns around health education delivery. Use of peers or expert clients in HIV settings has been adopted and provides useful lessons in this respect [25, 26].

Both providers and clients highlighted health system factors including long waiting time, inadequate logistics, shortage of personnel coupled with work over load, and space limitations. In their study carried out in Uganda over 5 years ago, Mutyaba and colleagues also highlighted similar health service factors as a deterrent to cervical cancer screening [12].

## Conclusion

In conclusion, the biggest barriers to screening were related to fears, misconceptions and lack of knowledge about cervical cancer and related services. These barriers could all be addressed with well-designed, context specific and rigorously tested cervical cancer education, targeted at both providers and clients to ensure that women understand the importance of what they are being offered, and the providers clearly understand their role in providing these services and can counsel women in a sensitive and effective way.

## Abbreviations

KI: Key informant
IDI: In-depth interview
HIV: Human Immune Deficiency Virus
ARVs: Antiretroviral drugs
WHO: World Health Organization
SRH: Sexual Reproductive Health
VIA: Visual Inspection with Acetic acid
VILI: Visual Inspection with Lugol’s Iodine

## References

[1] Lyimo FS, Tanya NB. Demographic, knowledge, attitudinal, and accessibility factors associated with uptake of cervical cancer screening among women in a rural district of Tanzania: Three public policy implications. BMC Public Health. Volume 12. Accessed on 30^th^ October, 2012 from http://www.ncbi.nlm.nih.gov/pubmed/22233530 10.1186/1471-2458-12-22 PMC3299640 22233530

[2] CDC. Cervical Cancer Screening for Women Who Attend STD Clinics or Have a History of STDs. Accessed on 26th May, 2012 from www.cdc.gov/std/treatment/2010/cc-screening.htm-

[3] Franceschi S, Jaffe H. Cervical Cancer Screening of Women Living with HIV Infection: A must in the Era of Antiretroviral Therapy. Clininical Infectious Diseases. 2007; 45(4):510–3. Epub 2007 Jul 5. Retrieved on December 20^th^ 2012 from http://cid.oxfordjournals.org/content/45/4/510.full 10.1086/520022 17638204

[4] Highleyman L. HPV in Women with HIV-Women and HIV: Human Papillomavirus’, the body. 2007 Retrieved on May 20^th^ 2012 from http://www.thebody.com/content/art42340.html

[5] Kahesa C, Mwaiselage J, Wabinga HR, Ngoma T, Kalyango J, Karamagi C, Association between invasive cancer of the cervix and HIV-1 infection in Tanzania: the need for dual screening. BMC Public Health. 2008 Retrieved on November 10^th^, 2012 from http://www.biomedcentral.com/1471-2458/8/262/abstract 10.1186/1471-2458-8-262 PMC2527006 18664298

[6] Apollinaire H, Antoine J, Didier KE, Badian, T, Patrick AC, Benjamin E EM, Albert M, Raoul M, Mamourou K, François D, Annie JS, The IeDEA West Africa collaboration. Cervical cancer screening by visual inspection in Côte d'Ivoire, operational and clinical aspects according to HIV status. BMC Public Health. 2012 Retrieved on 24th August 2014 from http://www.biomedcentral.com/1471-2458/12/237 10.1186/1471-2458-12-237 PMC3328262 22443255

[7] Kahesa C, Mwaiselage J, Wabinga HR, Ngoma T, Kalyango J, Karamagi C. Association between invasive cancer of the cervix and HIV-1 infection in Tanzania: the need for dual screening. BMC Public Health. Retrieved on November 10^th^, 2012 from http://www.biomedcentral.com/1471-2458/8/262/abstract 10.1186/1471-2458-8-262 PMC2527006 18664298

[8] WHO, ico. Human Papillomavirus and Related Cancers. Summary Report Update. September 15, 2010. UGANDA. Retrieved on January 4^th^ 2013 from http://s3.amazonaws.com/zanran_storage/www.who.int/ContentPages/82032421.pdf 10/01/2013

[9] Akinyemiju TF. Socio-Economic and Health Access Determinants of Breast and Cervical Cancer Screening in Low-Income Countries: Analysis of the World Health Survey. PLoS One. 2012;7(11):e48834. Published online 2012 November 14. doi:10.1371/journal.pone.0048834. Retrieved on 17^th^ June 2013 from http://www.ncbi.nlm.nih.gov/pmc/articles/PMC3498259/10.1371/journal.pone.0048834 PMC3498259 23155413

[10] Waller J, Bartoszek M, Marlow L, Wardle J (2009). Barriers to cervical cancer screening attendance in England: a population-based survey’. J. Med. Screen..

[11] Singh S, Badaya S. Factors influencing uptake of Cervical Cancer Screening among Women in India: A Hospital based Pilot Study. OMICS Publishing Group. 2012 Retrieved on October 30^th^, 2012 from http://www.omicsonline.org/factors-influencinguptake-of-cervical-cancer-screening-among-women-in-india-a-hospital-based-pilot-study-2161-0711.1000157.php?aid=7147

[12] Mutyaba T, Faxelid E, Mirembe F, Weiderpass. Influences on uptake of reproductive health services in Nsangi Community of Uganda and their implications for cervical cancer screening. Reproductive Health. 2007;4:4 doi:10.1186/1742-4755-4-4.Retrieved on November 2012 from http://www.reproductive-health-journal.com/content/4/1/4 10.1186/1742-4755-4-4 PMC1936416 17594474

[13] Kennedy CE, Spaulding AB, Brickley DB, Almers L, Mirjahangir J, Packel L, et al. Linking sexual and reproductive health and HIV interventions: a systematic review. J Int AIDS Soc. 2010;13:26. Retrieved on 10th October 2015 from http://www.ncbi.nlm.nih.gov/pubmed/?term=Brickley%20DB%5BAuthor%5D&cauthor=true&cauthor_uid=20642843 10.1186/1758-2652-13-26 PMC2918569 20642843

[14] Lindegren ML, Kennedy CE, Bain-Brickley D, Azman H, Creanga AA, Butler LM (2012). Integration of HIV/AIDS services with maternal, neonatal and child health, nutrition, and family planning services. Cochrane Database Syst Rev.

[15] Vo BN, Cohen CR, Smith RM, Bukusi EA, Onono MA, Schwartz K, et al. Patient satisfaction with integrated HIV and antenatal care services in rural Kenya. AIDS Care. 2012;24(11):1442-7. doi:10.1080/09540121.2011.652357. Epub 2012 Feb. Retrieved on 10th October 2015 from http://www.ncbi.nlm.nih.gov/pubmed/22296261] 10.1080/09540121.2011.652357 PMC3495002 22296261

[16] Liambila W, Askew I, Mwangi J,Ayisi R, Kibaru J, Mullick S. Feasibility and effectiveness of integrating provider-initiated testing and counselling within family planning services in Kenya. AIDS. 2009;23(Suppl 1):S115-21. doi: 10.1097/01.aids.0000363784.96321.43 .Retrieved on 10th October 2015 from http://www.ncbi.nlm.nih.gov/pubmed/20081383 10.1097/01.aids.0000363784.96321.43 20081383

[17] Fletcher FE, Buchberg M, Schover LR, Basen-Engquist K, Kempf MC, Arduino RC, Vidrine DJ. Perceptions of barriers and facilitators to cervical cancer screening among low-income, HIV-infected women from an integrated HIV clinic. AIDS Care. 2014;26(10):1229–35. doi:10.1080/09540121.2014.894617. Epub 2014 Mar 18. Accessed on 3^rd^ June 2015 from http://www.ncbi.nlm.nih.gov/pubmed/24635664 10.1080/09540121.2014.894617 PMC4087052 24635664

[18] Kumakech E, Andersson S, Wabinga H, Vanja Berggren V. Integration of HIV and cervical cancer screening perceptions and preferences of communities in Uganda. BMC Womens Health. 2015;15:23. Published online 2015 Mar 11. doi:10.1186/s12905-015-0183-4. Accessed on 3^rd^ June 2015 from http://www.ncbi.nlm.nih.gov/pmc/articles/PMC4359479/10.1186/s12905-015-0183-4 PMC4359479 25783655

[19] Rositch AF, Gatuguta A, Choi RY, Guthrie BL, Mackelprang RD, Bosire R, Manyara L, Kiarie JN, Smith JS, Farquhar C. knowledge and Acceptability of Pap smears, self –sampling and HPV vaccination among Adult Women in Kenya. PLoS One. 2012;7(7):e40766. Published online 2012 July 10. doi:10.1371/journal.pone.0040766 PMCID. Accessed on 18/12/2012 from http://www.ncbi.nlm.nih.gov/pmc/articles/PMC3393696/10.1371/journal.pone.0040766 PMC3393696 22808257

[20] Heather B, Claire T, Claire LT. Cervical Screening within HIV Care: Findings from an HIV-Positive Cohort in Ukraine. public library of science. PLoS One. 2012;7(4):e34708.Retrieved on 16^th^ June 2013 from http://www.ncbi.nlm.nih.gov/pmc/articles/PMC3335834/10.1371/journal.pone.0034706 PMC3335834 22545087

[21] Matovelo D, Magoma M, Rambau P, Massinde A, Masalu N. HIV serostatus and tumour differentiation among patients with cervical cancer at Bugando Medical Centre. BMC Res Notes. 2012 Accessed on December 20^th^ 2012 from http://www.biomedcentral.com/1756-0500/5/406 10.1186/1756-0500-5-406 PMC3502109 22862747

[22] Oliver CE, Chidinma VGO, Per Olof O, Karen OP. Willingness and acceptability of cervical cancer screening among HIV positive Nigerian women. BMC Public Health. 2013 Published online 2013 January 17. doi: 10.1186/1471-2458-13-46. Retrieved on 16^th^ June, 2013 from http://www.ncbi.nlm.nih.gov/pmc/articles/PMC3567931/10.1186/1471-2458-13-46 PMC3567931 23327453

[23] Hansen TB, Hukkelberg S, Haldorsen T, Heriksen T, Skare BG, Nygard M. Factors associated with non-attendance, opportunistic attendance and reminded attendance to cervical screening in an organized screening program: a cross-sectional study of 12,058 Norwegian women. BMC Public Health. 2011. Published online 2011 April 26. doi:10.1186/1471-2458-11-264. Retrieved on 16^th^ June 2013 from http://www.ncbi.nlm.nih.gov/pmc/articles/PMC3111379/10.1186/1471-2458-11-264 PMC3111379 21521515

[24] Chizoma MN, Bola AO Awareness, perception and factors affecting utilization of cervical cancer screening services among women in Ibadan, Nigeria: a qualitative study. BMC- Reproductive Health. Reprod Health. 2012;9:11. Published online 2012 August 6. doi: 10.1186/1742-4755-9-11. Retrieved on 16^th^ June 2013 from http://www.ncbi.nlm.nih.gov/pmc/articles/PMC3539913/10.1186/1742-4755-9-11 PMC3539913 22866676

[25] Katzenstein D, McFarland W, Mbizvo M, Latif A, Machekano R, Parsonnet J (1998). Peer education among factory workers in Zimbabwe: providing a sustainable HIVprevention intervention.

[26] Gifford A, Laurent D, Gonzales V, Chesney M, Lorig K (1998). Pilot of randomized trial of education to improve self-management skills to men with symptomatic HIV/AIDS. J. Acquir. Immune Defic. Syndr. Hum. Retrovirol..

